# Long-Range Interactions Between Neighboring Nanoparticles Tuned by Confining Membranes

**DOI:** 10.3390/nano15120912

**Published:** 2025-06-12

**Authors:** Xuejuan Liu, Falin Tian, Tongtao Yue, Kai Yang, Xianren Zhang

**Affiliations:** 1 Langfang Key Laboratory of Cell Engineering and Applied Research, Langfang Key Laboratory of Food Nutrition and Safety, Biology Experimental Teaching Demonstration Center, Technical Innovation Center for Utilization of Edible and Medicinal Fungi, College of Life Science, Langfang Normal University, Langfang 065000, China; 1231941@lfnu.edu.cn; 2 Chinese Academy of Sciences Center for Excellence in Nanoscience National Center for Nanoscience and Technology, Beijing 100190, China; tianfl@nanoctr.cn; 3 Institute of Coastal Environmental Pollution Control, Key Laboratory of Marine Environment and Ecology (Ministry of Education), Frontiers Science Center for Deep Ocean Multispheres and Earth System, Ocean University of China, Qingdao 266100, China; yuetongtao@ouc.edu.cn; 4 Center for Soft Condensed Matter Physics and Interdisciplinary Research , School of Physical Science and Technology, Soochow University, Suzhou 215006, China; 5 State Key Laboratory of Organic-Inorganic Composites, Beijing University of Chemical Technology, Beijing 100029, China

**Keywords:** nanoparticles, confining membrane, long-range interactions, dissipative particle dynamics simulations

## Abstract

Membrane tubes, a class of soft biological confinement for ubiquitous transport intermediates, are essential for cell trafficking and intercellular communication. However, the confinement interaction and directional migration of diffusive nanoparticles (NPs) are widely dismissed as improbable due to the surrounding environment compressive force. Here, combined with the mechanics analysis of nanoparticles (such as extracellular vesicles, EVs) to study their interaction in confinement, we perform dissipative particle dynamics (DPD) simulations to construct a model that is as large as possible to clarify the submissive behavior of NPs. Both molecular simulations and mechanical analysis revealed that the interactions between NPs are controlled by confinement deformation and the centroid distance of the NPs. When the centroid distance exceeds a threshold value, the degree of crowding variation becomes invalid for NPs motion. The above conclusions are further supported by the observed dynamics of multiple NPs under confinement. These findings provide new insights into the physical mechanism, revealing that the confinement squeeze generated by asymmetric deformation serves as the key factor governing the directional movement of the NPs. Therefore, the constraints acting on NPs differ between rigid confinement and soft confinement environments, with NPs maintaining relative stillness in rigid confinement.

## 1. Introduction

Many studies address transmembrane transport but ignore barriers for cargo delivery into complex and confined space [1,2,3,4,5,6]. Biologically, membrane tubes are ubiquitous transport intermediates in a variety of cellular activities, which are essential for membrane area storage [7], cell trafficking, and intercellular communication [8,9,10,11,12,13]. They have high aspect ratios, extremely high curvatures, and small radial dimensions, and they are dynamic structures [14,15]. Numerous theoretical and experimental investigations have focused on the mechanisms of tubular formation and deformation, including phenomena such as membrane tube pearling [16]. However, limited research has addressed the underlying mechanism governing tubular delivery in detail. The precise physical mechanism driving the versatile movement of cargo within such confined structures remains insufficiently understood. In particular, the influence of membrane rigidity of the intracellular crowded environment and narrow intercellular junctions on particle delivery, which is determined by a combination of the following factors [17,18], remains an unresolved mechanistic question: (1) the molecular composition, such as phospholipid saturation, cholesterol content, or membrane protein distribution; (2) dynamic structural response, such as membrane curvature elasticity or phase separation behavior.

Both in vivo and in vitro, experimental results have shown that organelles, proteins, colloidal particles, DNA, bacteria, or virus particles can be transported between cells through membrane tubes [19,20,21,22]. The transfer of material and information of bacteria is also finished by membrane tubes [23]. In particular, the transmission of some common viruses, such as pathogens, is increased by tubes, including human immunodeficiency virus (HIV)-1 in vivo and prions in vitro [20]. However, the substance transported will be larger than the tube; for example, the radius of the gyrase for the DNA molecule of the T7-phage is ~560 nm, and the diameter of the tube is generally approximately a few hundred nanometers [24]. Most of these natural transport vectors are nanometer-sized. Moreover, with the development of nanotechnology, nanoparticles (NPs) have become one of the promising vehicles for targeted delivery [25,26,27]. Diffusion transport of NPs in intracellular crowded environments is critical to the efficiency of delivery into cells, particularly, as is well known, the interactions among multiple nanoparticles within a membrane tube during co-endocytosis [28]. Therefore, the unidentified interactions involved in nanoparticle transport within narrow confinement have prompted investigation into the physical mechanisms regulating their movement.

In the present study, we focus on analyzing how confined space compression alters interparticle forces. To establish a universal mechanism for large-scale molecular dynamics simulations, a simplified model is constructed using two flat membranes to form a confined space, with vesicles serving as representations of the transported contents. We examine the compression process along one symmetric direction of 3D narrow intercellular junctions or intracellular crowded environments, which is shown to have negligible impact on the research conclusions. The confinement squeezing force is characterized by the effective separation between the two membranes (*H*_eff_) [29]_,_ which determines the confinement size. And when the effective separation distance of the two membranes (*H*_eff_) becomes smaller than the diameter of the vesicles, confinement is applied in the simulation, generating a squeezing force on the vesicles. Moreover, this simulation system enables convenient and precise observation of the dynamic behavior of particles within a confined environment.

## 2. Computational Details

Here, we employed the dissipative particle dynamics (DPD) method to compute a simulation system as large as possible [30,31,32,33]. In this method, the dynamics of each elementary unit are governed by Newton’s equation of motion, ($\frac{{dr}_{i}}{dt}=v_{i};\frac{{dv}_{i}}{dt}=f_{i}/m_{i})$, which includes a short-range repulsive force$\;f_{i}$ consisting of a conservative force ($F_{ij}^{C}$), a dissipation force ($F_{ij}^{D}$), and a random force ($F_{ij}^{R}$). This method is similar to molecular dynamics (MD). In particular, the conservative force is given by $F_{ij}^{C}=\left\{\begin{array}{c} a_{ij}\left(1-r_{ij}\right)\tilde{r_{ij}}\;r_{ij}<r_{c}\\ \;0\;r_{ij}\geq r_{c} \end{array}\right.$, where $a_{ij}$ is the maximum repulsive strength between beads *i* and *j*, $r_{ij}=r_{j}-r_{i}\;(r_{i}\;and\;r_{j}\;$are the positions of beads *i* and *j*), and ${\tilde{r}}_{ij}=\left|r_{ij}\right|/r_{ij}$. Typically, we set the cutoff radius $r_{c}$, the bead mass *m*, and the thermostat temperature $k_{B}T$ to unite our simulations and in the following description. The dissipation force is calculated by $F_{ij}^{D}=-\gamma \left(1-\frac{r_{ij}}{r_{c}}\right)2({\tilde{r}}_{ij}\cdot v_{ij}){\tilde{r}}_{ij}$, where γ is the friction coefficient and where $v_{ij}=v_{j}-v_{i}$ ($v_{i}$ and $v_{j}$ are the bead velocities). The random force formula is $F_{ij}^{R}=-\sigma \left(1-\frac{r_{ij}}{r_{c}}\right)2\theta_{ij}{\tilde{r}}_{ij}$, where σ is the noise amplitude and where $\theta_{ij}$ is an uncorrelated random variable with zero mean and unit variance.

The soft beads are derived from the vesicle model commonly employed in the DPD method, and the bond between adjacent beads is governed by a harmonic spring force, defined as $F_{S}=K_{S}\left(r_{ij}-r_{eq}\right){\tilde{r}}_{ij}$, where $K_{S}$ and $r_{eq}$ are the spring constant and the equilibrium bond length, respectively. The values in the simulation are set to 128$k_{B}T$ and 0.45${\;r}_{c}$. The force constraining the variation in the bond angle is given by $F_{\varphi }=-\nabla U_{\varphi }$ and $U_{\varphi }=K_{\varphi }(1-\cos\left(\varphi -\varphi_{0})\right)$, which are used to maintain the bending rigidity of the lipids. The equilibrium bending angle is $\varphi_{0}=\pi$, and the bond bending force constant is $K_{\varphi }=10.0k_{B}T$. With different simulation environments, the sizes of the simulation boxes are $150\times 45\times 80\;r_{c}^{3}$ and $220\times 45\times 80\;r_{c}^{3}$. Periodic boundary conditions were implemented in all three directions. In the simulation, *a_ij_* represented the interaction parameters of the DPD beads, which were set to *a_hh_* = *a_tt_* = *a_ww_* = *a_hw_* = 25 and *a_ht_* = *a_tw_* = 200. The subscript of *a_ij_* denotes different types of beads in the system: *h* and *t* represent the lipid head and tail for the membrane and vesicle, and *w* is for water beads.

**Figure 1:**
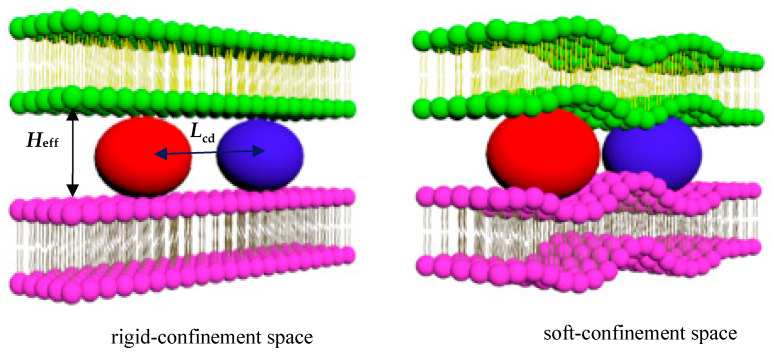
Initial configuration of the simulation system: rigid confinement space and soft confinement space. The vesicle model represents soft particle transport in confinement: *H*_eff_ represents the effective separation between the two membranes, and *L*_cd_ represents the centroid distance between two vesicles.

The simulation model consists of vesicles confined between two lipid bilayers, where the variation in bilayer rigidity represents either a rigid confinement or soft confinement environment (see Figure 1). When the effective separation distance (*H*_eff_) becomes smaller than the diameter of the vesicles, confinement is applied in the simulation, generating a squeezing force on the nanoparticles (vesicles). This force regulates the centroid distance (*L*_cd_) between the two vesicles. In the simulation, the effective distance *H*_eff_ is calculated as $H_{eff}=N/(3\times l_{x}\times l_{y})$, where *N* is the number of water beads inside the confined space and *l_x_* and *l_y_* correspond to the dimensions of the simulation box in the *x* and *y* directions, respectively. Therefore, the squeezing force produced by the confined space is controlled by the number of water beads inside or outside the membrane. Particular attention is given to the initial model in the simulation. To ensure the random distribution of water beads while avoiding overlap with lipid molecules, the spatial arrangement of the initial solvent molecules was manually configured, resulting in a non-equilibrium system. The first essential step involved equilibrating the solvent beads. To achieve this, random initial velocities were assigned to the solvent molecules, allowing the system to evolve and reach equilibrium within the simulation box over a sufficiently long time step. The coordinates of all the particles in the simulated system were the new initial configuration for the next formal simulation calculation.

## 3. Results and Discussion

### 3.1. Directional Movement of Neighboring Vesicles Tuned by Confining Membranes

**Figure 2:**
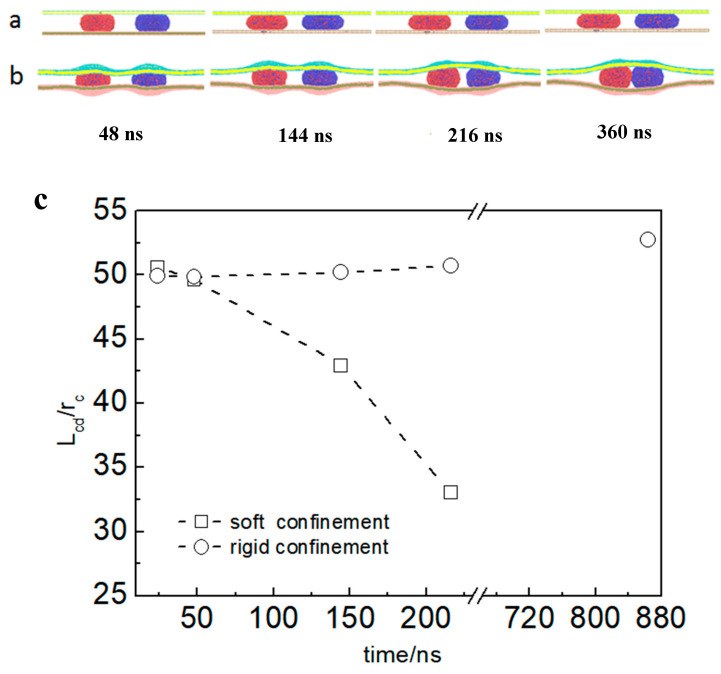
Different pathways of vesicle transport in soft confinement and rigid confinement. (**a**,**b**) Typical snapshots of the vesicle transport process: (**a**) rigid confinement *H*_eff_ = 12 *r*_c_, *L*_init-cd_ = 50 *r*_c_; (**b**) soft confinement *H*_eff_ = 12 *r*_c_, *L*_init-cd_ = 50 *r*_c_. (*L*_init-cd_ is the initial distance between the vesicles in confinement); (**c**) comparison of the time evolution of *L*_cd_ (the distance between the vesicles in confinement).

To directly examine the differences in vesicle movement between rigid and soft confined spaces, simulations were first conducted using two systems that differed only in the rigidity of the confined space. In the rigid confinement system, the membrane shape remained fixed, whereas in the soft confinement system, the membrane was capable of deformation through self-adaptive regulation during vesicle interactions. The time evolution of the typical dynamic process of vesicle movement is shown in Figure 2a,b. Simulation snapshots revealed that the deformation of the membrane implied an attractive force between the vesicles (Figure 2b). However, the vesicles in the rigid confinement zone had barely moved. *L*_cd_ is almost a straight line, and the simulation time is more than 840 ns (see Figure 2c). The figure also indicates that *L*_cd_ gradually decreases in soft confinement. Using this simplified model, two contrasting responses were observed for vesicle movement within the confined space. Specifically, deformation in soft confinement appeared to play a significant role in promoting vesicle movement, whereas in rigid confinement, where deformation was absent, the vesicles remained stationary.

To further understand the special interactions between carriers (such as vesicles) in confinement, we systematically studied the directional movement of vesicles in different simulated environments. The two primary variables, the size of the confined space (*H*_eff_) and the initial centroid distance between two vesicles (*L*_init-cd_), were adjusted for the model.

**Figure 3:**
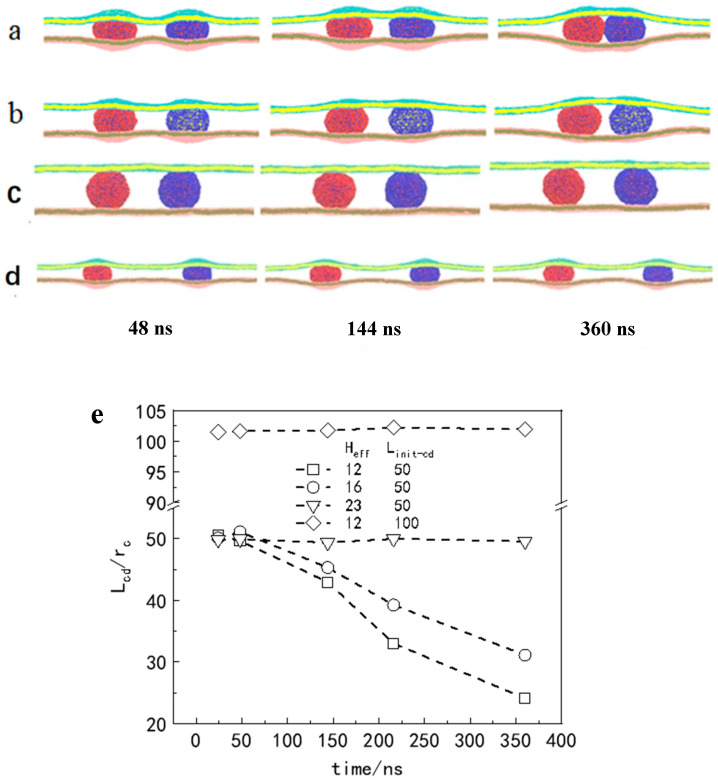
Effect of the initial effective separation between the two membranes (*H*_eff_) and the initial distance between the mass centers of the two vesicles (*L*_init-cd_) on the transport of vesicles in soft confinement. (**a**–**d**) Typical dynamic process of vesicle transport under different conditions: (**a**) *H*_eff_ = 12 *r*_c_, *L*_init-cd_ = 50 *r*_c_; (**b**) *H*_eff_ = 16 *r*_c_, *L*_init-cd_ = 50 *r*_c_; (**c**) *H*_eff_ = 23 *r*_c_, *L*_init-cd_ = 50 *r*_c_; (**d**) *H*_eff_ = 12 *r*_c_, *L*_init-cd_ = 100 *r*_c_. (**e**) shows the time evolution of the *L*_cd_ variation between the vesicles under different confinement conditions.

To determine how *H*_eff_ and *L*_init-cd_ cooperate to determine the consequences of vesicle interactions in soft confinement, namely, motorial vesicles or motionless vesicles, we first compared four different situations, including *H*_eff_ = 12 *r*_c_, *H*_eff_ = 16 *r*_c_, and *H*_eff_ = 23 *r*_c_, while *L*_init-cd_ was fixed at 50 *r_c_*. Note that the radius for the counterpart of the vesicle in confinement is controlled by the given number of encapsulated solvent beads, Nv-in (R_v-in_ = 14.3 r_c_ for N_v-in_ = 14,000). The corresponding dynamic pathways are shown in Figure 3a–c. As demonstrated across varying sizes of confined space, vesicle movement was initiated by confinement deformation, with smaller confinement resulting in increased movement speed (see Figure 3e). As illustrated in Figure 3d,e, minimal open space was found to be insufficient in effectively driving vesicle movement. It seems that vesicle trafficking is predominantly governed by confinement–deformation coupling. However, we also found that as long as the center of mass distance exceeds a critical value (*L*_init-cd_ = 100 *r*_c_), two vesicles basically remain relatively static, even when they are subjected to strong compression (*H*_eff_ = 12 *r*_c_) in a confined space (see Figure 3d,e).

**Figure 4:**
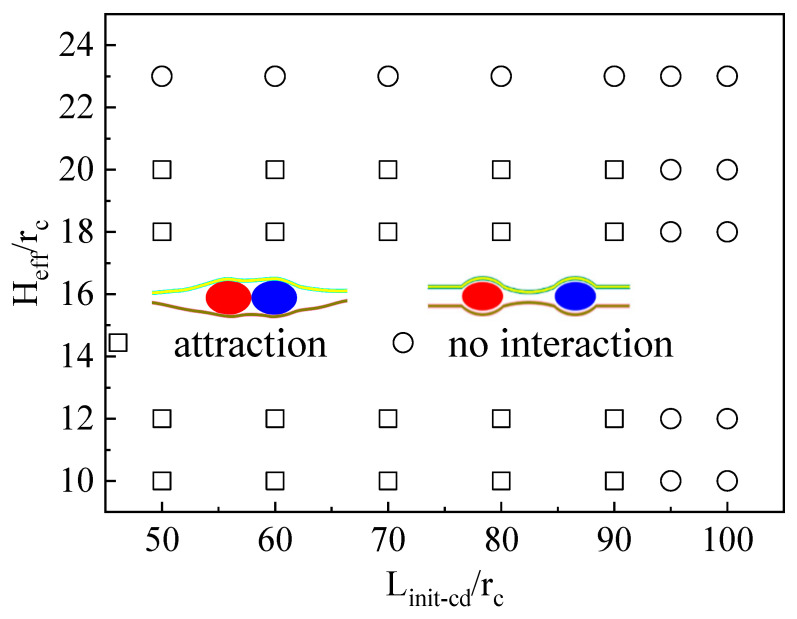
Phase diagram of vesicle transport in soft confinement as a function of *H_eff_* and *L_init-cd_*.

The laws for vesicle movement in confinement can be summarized as follows. When the initial centroid distance of the two vesicles (*L*_init-cd_) is unchanged, weakening the compression of the confined space does not result in noninteraction between the vesicles. A certain deformation of the confined space induces the carriers (such as vesicles) to be close to each other, resulting in an attractive force. The *L*_init-cd_ between vesicles determines whether the vesicles have direct motion. To further prove this conclusion, the vesicle interaction and the motion of the vesicle in a soft confined space were systematically summarized, where *H*_eff_ and *L*_init-cd_ were changed (see Figure 4). When the *L*_init-cd_ between vesicles was greater than ~95 *r*_c_, the vesicles did not interact in this case regardless of how crowded the space was.

### 3.2. The Confinement of Asymmetric Deformation Causes Long-Range Interactions Between Neighboring Vesicles

**Figure 5:**
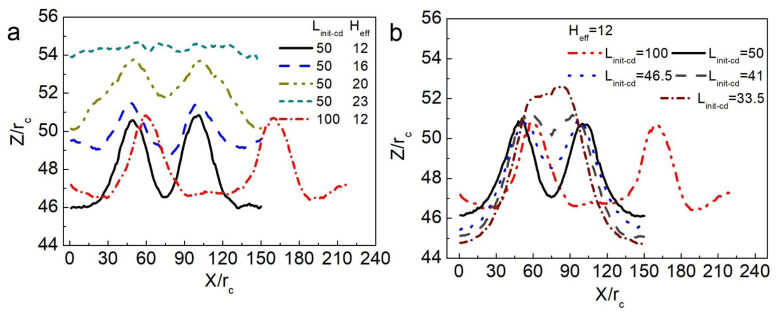
(**a**) Deformation of the upper confinement membrane along the Z direction at the simulation time of *t =* 48 ns. (**b**) The vesicles are frozen, and the final state deformation of the upper confinement membrane with different *L*_init-cd_ variations along the Z direction.

We analyzed the confinement deformation in detail to describe the effect on vesicle movement. When the *L*_init-cd_ between two vesicles was unchanged, the *H*_eff_ in the confined space was gradually reduced, namely, the squeezing force of the confined space on the vesicle increased, and we observed more obvious deformation of the confined space, as shown in Figure 5a. In energetic terms, the deformation energy of the system increased. As a result, only the position of the vesicles in the system being changed could reduce the increased deformation energy. As *H*_eff_ increased, the simulation results clearly revealed that the confinement squeezing force decreased and that the interaction between vesicles weakened. A comparison of Figure 5a,b reveals that when two vesicles were in the same confined space (*H*_eff_ = 12 *r*_c_), and the *L*_init-cd_ of the vesicle was 50 *r_c_* or 100 *r*_c_, the confinement deformation was quite different. When *H*_eff_ was fixed, which gradually reduced the *L*_init-cd_ of the two vesicles, the curve in Figure 5b shows that the deformation of the confined space in the middle of the two vesicles decreased, whereas the bulge of a curve of confinement deformation on both sides of the vesicle increased in size. Moreover, the results in Figure 3d and Figure 4 show that two vesicles with *L*_init-cd_ = 100 *r*_c_ basically remained stationary. We believe that the essence of the above phenomenon can be attributed to the following reasons.

As the *L*_init-cd_ between the two vesicles decreased, the deformation of the confined space in the middle of the two vesicles decreased compared with the deformation on both sides (the left side of the left vesicle and the right side of the right vesicle) (see Figure 5b). As is well understood, the deformation of each vesicle aligns with that of the surrounding confined space. Specifically, the left-side deformation of the left vesicle and the right-side deformation of the right vesicle were both smaller than their corresponding opposite-side deformations. This discrepancy indicates that the symmetry of membrane deformation within the soft confined space on either side of each vesicle was disrupted. With decreasing *L*_init-cd_, the confinement deformation broken symmetry became increasingly larger, making the vesicle deformation symmetry breaking in the confined space more distinct. We believe that this asymmetric deformation contributed to directional vesicle movement.

### 3.3. Force Analysis of Vesicles in Soft Confined Space

**Figure 6:**
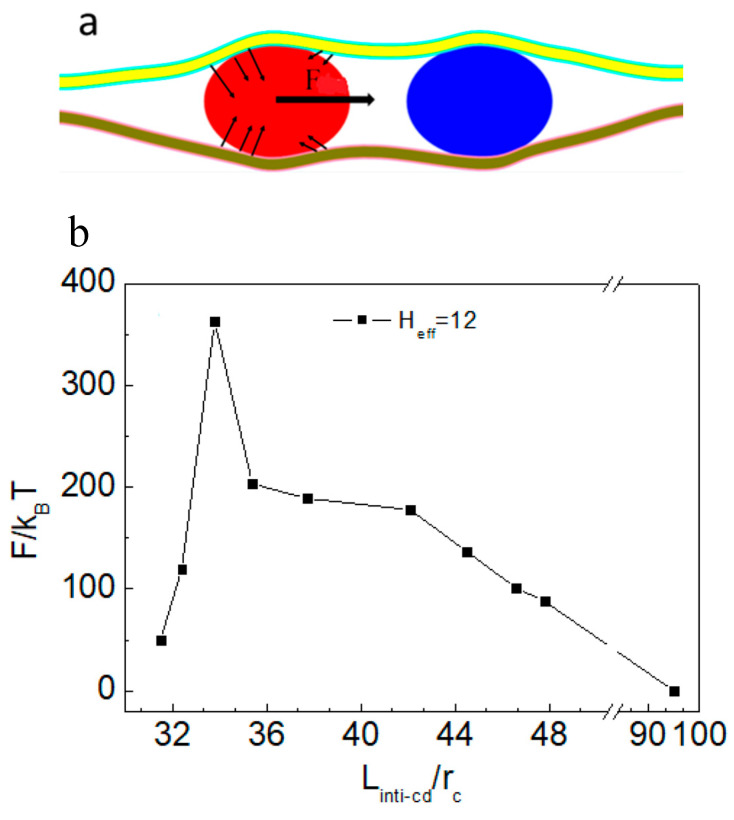
(**a**) Schematic diagram showing the interaction between two neighboring nanoparticles (*F*) induced by the asymmetric deformation of soft confinement. (**b**) The horizontal component of the resultant force *F* as a function of the initial distance between the two vesicles. In this figure, *H*_eff_ = 12 *r*_c_.

According to the above discussion, when the vesicle is squeezed, the direction of the squeezing force (from soft confinement) at each point on the vesicle surface membrane changes. Therefore, we propose that the asymmetry in confinement deformation can induce a directional component of force (*F*) along the line connecting the centers of mass of the vesicles, thereby mediating their relative motion (Figure 6a).

In the simulation, the force acting on the left vesicle in the confined space was systematically calculated (Figure 6b). As shown in Figure 5b, decreasing the *L_cd_* between vesicles increased the asymmetry of the confined space, thereby enhancing the directional squeezing force exerted on the vesicle. As indicated in Figure 6b, when the *L*_cd_ of two vesicles was less than or equal to 50 *r*_c_, the F value of the left vesicle in the system was negative (the two vesicles had an attractive effect). With decreasing *L*_init-cd_ between vesicles, the squeezing force on the vesicles gradually increased. When *L*_cd_ was ~33.5 *r*_c_, F reached the minimum value. However, F starts to decrease when *L*_cd_ is less than ~33.5 *r*_c_. Figure 5b shows that the bending deformation of the membrane in soft confinement between vesicles almost disappeared at *L_cd_* ~33.5 *r*_c_. It is speculated that the deformation energy of the system gradually approaches the minimum value at this time and that the deformable interaction-mediated vesicle motion weakens. When *L_cd_* between two vesicles exceeds 95 *r*_c_, the vesicles remain relatively stationary (Figure 4). Therefore, we next calculate the force on the left vesicle in the soft confined space with *H*_eff_ = 12 *r*_c_ and *L*_cd_ = 100 *r*_c_. After 1,000,000 steps of balance computation, the force of the vesicle was found to be ~0 *k*_B_*T/r*_c_. Therefore, as long as *L_cd_* exceeds a critical value, soft confinement deformation cannot mediate vesicle interactions to achieve directional movement. It is easy to infer from the geometric relationship that the deformation of the confined space is basically symmetric, with a sufficiently large numeric value of *L*_cd_.

The above results indicate that the motion between particles in crowded environments is very sensitive to the asymmetry of confinement deformation. The important parameter related to this deformation is further proven via force analysis, i.e., the centroid distance between particles in a crowded environment. We can change the center of mass distance between the particles to adjust their interaction in the crowded environment, thus controlling their motion.

### 3.4. Motion of Multiple Particles in a Soft Confined Space

The transfer of materials or information between adjacent cells often requires the coordinated movement of multiple substances to ensure continuous delivery. Consequently, gaining deeper insights into the collective motion of multiple particles within confined environments is crucial for understanding cellular activities in living organisms. To address this problem, two typical deformation symmetry-breaking models were established by squeezing three or four vesicles into soft confinement. Owing to the limitations of the simulation scale, we researched only the movement of vesicles with a small *L*_init-cd_, where the vesicles exhibit mutual attraction.

**Figure 7:**
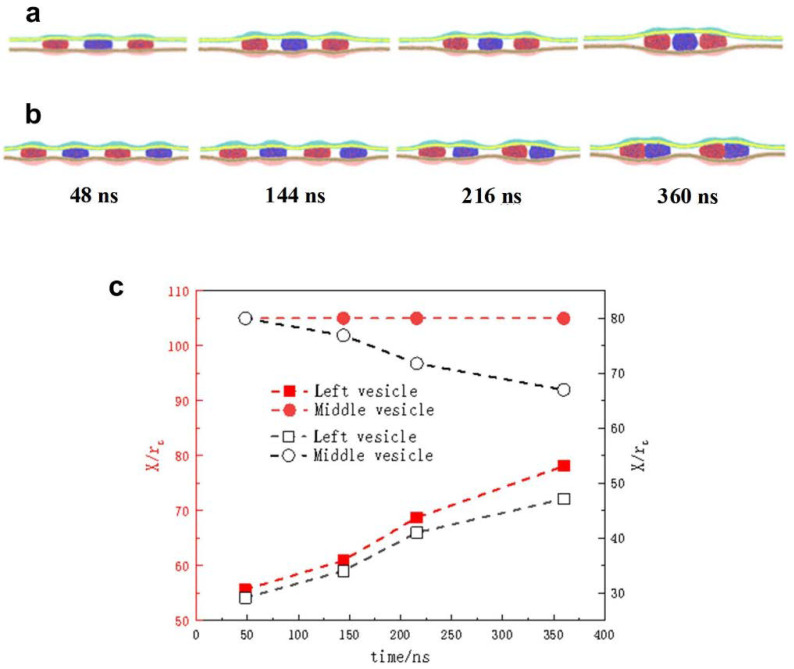
(**a**,**b**) Typical dynamic snapshots of three or four vesicles in confinement: *H*_eff_ = 12 *r*_c_, *L*_init-cd_ = 50 *r*_c_. (**c**) The position of the vesicles varies as a function of the simulation time, and the vertical coordinate represents the center-of-mass coordinate of the vesicle in the x direction: the red color represents three vesicles in the confined space, and the black color represents four vesicles in the confined space.

The dynamics of the three vesicles in the confined space were observed in detail (Figure 7a). The vesicles on both sides moved toward the middle vesicle, while the middle vesicle remained stationary. As evidenced by the preceding analysis and conclusions, the vesicles positioned at both ends were gradually squeezed toward the central vesicle due to the asymmetric deformation of the soft confinement. Positioned equidistant from the left and right boundaries, the middle vesicle resided in a symmetrically deformed region of the confinement, resulting in a net force acting on it that was near zero. Eventually, these three vesicles gathered together. In Figure 7c, the position analysis of *L*_cd_ also further illustrates the results of vesicle movement.

When four vesicles were placed within a confinement possessing the same *L*_init-cd_, the central vesicles initially experienced a symmetrically deformed environment, while the two vesicles near the boundaries were subjected to asymmetrical confinement deformation. As a result, the edge vesicles gradually moved toward the central ones. Once this movement was initiated, the symmetric deformation around the central vesicles was disrupted, generating forces directed toward the ends of the confinement. These forces acted on the central vesicles, causing them to move apart and align with the approaching edge vesicles, respectively (see Figure 7b). As shown in Figure 7c, the change in *L*_cd_ is consistent with the vesicle dynamics. The figure curves show that the centroid coordinate x of the outermost left vesicle gradually increased with increasing simulation time, whereas the value of the other vesicle beside it decreased. Therefore, the vesicles ultimately paired together. The dynamic analysis of multiple vesicles further supports our conclusion that asymmetrical deformation of the confinement governs the interactions between vesicles, driving their directional motion. This process ultimately leads to the minimization of the system’s deformation energy.

We found that the directional delivery of vesicles in the confined space was related to *L*_cd_, revealing a simple and important mechanical mechanism in which confinement asymmetry deformation could control the interactions between particles.

## 4. Conclusions

When nanoparticles are delivered into confined regions commonly encountered in biological systems, they experience squeezing forces that differ from those observed in either rigid or soft confinement environments. In the present study, when the centroid distance of NPs is less than a critical value (*L*_init-cd_~95 *r*_c_), they exhibit interparticle attraction, leading to directional aggregation. We found that asymmetric deformation within the crowded environment facilitated the directional movement of the particles. Long-range attractive interactions occur ubiquitously in intracellular crowded environments and narrow intercellular junctions, and we expect our work could provide an explanation for some experimental observations of vesicle-oriented transport. This mechanical mechanism allows us to further understand some interesting phenomena of interparticle transport and transmission in the complex physiological environment of living organisms.

## Data Availability

The data presented in this study are available from the corresponding author upon reasonable request.

## References

[1] Chen P., Yue H., Zhai X., Huang Z., Ma G., Wei W., Yan L. (2019). Transport of agraphene nanosheet sandwiched inside cell membranes. Sci. Adv..

[2] Liu Y., Li S., Liu X., Sun H., Yue T., Zhang X., Yuan B., Cao D. (2019). Design of Small Nanoparticles Decorated with Amphiphilic Ligands: Self-Preservation Effect and Translocation into a Plasma Membrane. ACS Appl. Mater. Interfaces.

[3] Foroozandeh P., Aziz A.A. (2018). Insight into cellular uptake and intracellular trafficking of nanoparticles. Nanoscale Res. Lett..

[4] Tajima M., Nakamura H., Ohsaki S., Watano S. (2024). Effect of cholesterol on nanoparticle translocation across a lipid bilayer. Phys. Chem. Chem. Phys..

[5] Mitchell M.J., Billingsley M.M., Haley R.M., Wechsler M.E., Peppas N.A., Langer R. (2021). Engineering precision nanoparticles for drug delivery. Nat. Rev. Drug Discov..

[6] Zhang S., Gao H., Bao G. (2015). Physical principles of nanoparticle cellular endocytosis. ACS Nano.

[7] Davis D.M., Sowinski S. (2008). Membrane nanotubes: Dynamic long-distance connections between animal cells. Nat. Rev. Mol. Cell Biol..

[8] Sharma S., Masud M.K., Kaneti Y.V., Rewatkar P., Koradia A., Hossain M.S.A., Yamauchi Y., Popat A., Salomon C. (2021). Extracellular vesicle nanoarchitectonics for novel drug delivery applications. Small.

[9] Hossen S., Hossain M.K., Basher M.K., Mia M.N.H., Rahman M.T., Uddin M.J. (2019). Smart nanocarrier-based drug delivery systems for cancer therapy and toxicity studies: A review. J. Adv. Res..

[10] Sedgwick A.E., D’Souza-Schorey C. (2018). The biology of extracellular microvesicles. Traffic.

[11] Sowinski S., Jolly C., Berninghausen O., Purbhoo M.A., Chauveau A., Köhler K., Oddos S., Eissmann P., Brodsky F.M., Hopkins C. (2018). Membrane Nanotubes Physically Connect T Cells over Long Distances Presenting a Novel Route for HIV-1 Transmission. Nat. Cell Biol..

[12] Wang Z.G., Liu S.L., Tian Z.Q., Zhang Z.L., Tang H.W., Pang D.W. (2012). Myosin-Driven Intercellular Transportation of Wheat Germ Agglutinin Mediated by Membrane Nanotubes Between Human Lung Cancer Cells. ACS Nano.

[13] Chauveau A., Aucher A., Eissmann P., Vivier E., Davis D.M. (2010). Membrane Nanotubes Facilitate LongDistance Interactions Between Natural Killer Cells and Target Cells. Proc. Natl. Acad. Sci. USA.

[14] Simunovic M., Evergren E., Golushko I., Prévost C., Renard H., Johannes L., McMahon H.T., Lorman V., Voth G.A., Bassereau P. (2016). How curvature-generating proteins build scaffolds on membrane nanotubes. Proc. Natl. Acad. Sci. USA.

[15] Noguchi H., Tozzi C., Arroyo M. (2022). Binding of anisotropic curvature-inducing proteins onto membrane tubes. Soft Matter.

[16] Liu X., Tian F., Yue T., Zhang X., Zhong C. (2016). Exploring the shape deformation of biomembrane tubes with theoretical analysis and computer simulation. Soft Matter.

[17] Karal M.A.S., Billah M.M., Ahmed M., Ahamed M.K. (2023). A review on the measurement of the bending rigidity of lipid membranes. Soft Matter.

[18] Lee T.H., Charchar P., Separovic F., Reid G.E., Yarovsky I., Aguilar M.-I. (2024). The intricate link between membrane lipid structure and composition and membrane structural properties in bacterial membranes. Chem. Sci..

[19] Rustom A., Saffrich R., Markovic I., Walther P., Gerdes H.H. (2004). Nanotubular highways for intercellular organelle transport. Science.

[20] Hurtig J., Chiu D.T., Önfelt B. (2010). Intercellular nanotubes: Insights from imaging studies and beyond. WIREs Nanomed. Nanobiotechnol..

[21] Epperla C.P., Mohan N., Chang C.W., Chen C.-C., Chang H.C. (2016). Nanodiamond-mediated intercellular transport of proteins through membrane tunneling nanotubes. Small.

[22] Onfelt B., Davis D.M. (2004). Can membrane nanotubes facilitate communication between immune cells?. Biochem. Soc. Trans..

[23] Dubey G.P., Benyehuda S. (2011). Intercellular nanotubes mediate bacterial communication. Cell.

[24] Karlsson A., Karlsson R., Karlsson M., Cans A.S., Strömberg A., Ryttsén F., Orwar O. (1999). Molecular engineering: Networks of nanotubes and containers. Nature.

[25] Khan F.A., Albalawi R., Pottoo F.H. (2022). Trends in targeted delivery of nanomaterials in colon cancer diagnosis and treatment. Med. Res. Rev..

[26] Marquez C.A., Oh C.I., Ahn G., Shin W.R., Kim Y.H., Ahn J.Y. (2024). Synergistic vesicle-vector systems for targeted delivery. J. Nanobiotechnol..

[27] Zhao S., Di Y., Fan H., Xu C., Li H., Wang Y., Wang W., Li C., Wang J. (2024). Targeted delivery of extracellular vesicles: The mechanisms, techniques and therapeutic applications. Mol. Biomed..

[28] Xiong K., Zhao J., Yang D., Cheng Q., Wang J., Ji H. (2017). Cooperative wrapping of nanoparticles of various sizes and shapes by lipid membranes. Soft Matter.

[29] Liu X., Tian F., Yue T., Zhang X., Zhong C. (2017). Pulling force and surface tension drive membrane fusion. J. Chem. Phys..

[30] Groot R.D., Warren P.B. (1997). Dissipative particle dynamics: Bridging the gap between atomistic and mesoscopic simulation. J. Chem. Phys..

[31] Sreekumari A., Lipowsky R. (2018). Lipids with bulky head groups generate large membrane curvatures by small compositional asymmetries. J. Chem. Phys..

[32] Li C., Tang Y., Lin H., Zhang C., Liu Z., Yu L., Wang X., Lin Y. (2023). Novel multiscale simulations on the membrane formation via hybrid induced phase separation process based on dissipative particle dynamics. Sep. Purif. Technol..

[33] Mitsuhashi H., Morikawa R., Noguchi Y., Takasu M. (2023). Dissipative particle dynamics simulations for shape change of growing lipid bilayer vesicles. Life.

